# Breathlessness is associated with urinary incontinence in men: A community-based study

**DOI:** 10.1186/1471-2466-10-2

**Published:** 2010-01-06

**Authors:** Fumi Hirayama, Andy H Lee, Tetsuo Hiramatsu, Yoshimasa Tanikawa

**Affiliations:** 1 School of Public Health, Curtin Health Innovation Research Institute, Curtin University of Technology, Perth, WA, Australia; 2 Department of Respiratory Medicine and Clinical Immunology, Toyota Kosei Hospital, Aichi Prefectural Welfare Federation of Agricultural Cooperatives, Aichi, Japan; 3 Department of Respiratory Medicine and Allergy, Komaki City Hospital, Aichi, Japan

## Abstract

**Background:**

Urinary incontinence (UI) is a distressing problem for older people. To investigate the relationship between UI and respiratory symptoms among middle-aged and older men, a community-based study was conducted in Japan.

**Methods:**

A convenience sample of 668 community-dwelling men aged 40 years or above was recruited from middle and southern Japan. The International Consultation on Incontinence Questionnaire-Short Form, the Medical Research Council's dyspnoea scale and the Australian Lung Foundation's Feeling Short of Breath scale, were administered by face-to-face interviews to ascertain their UI status and respiratory symptoms.

**Results:**

The overall prevalence of UI was 7.6%, with urge-type leakage (59%) being most common among the 51 incontinent men. The presence of respiratory symptoms was significantly higher among incontinent men than those without the condition, especially for breathlessness (45% versus 30%, *p *= 0.025). The odds of UI for breathlessness was 2.11 (95% confidence interval 1.10-4.06) after accounting for age, body mass index, smoking and alcohol drinking status of each individual.

**Conclusions:**

The findings suggested a significant association between UI and breathlessness in middle-aged and older men.

## Background

Urinary incontinence (UI) is a distressing condition and costly problem for older adults [1]. Established risk factors of UI are age, gender, obesity and smoking [2-4]. The prevalence of UI is also known to be higher among women with cystic fibrosis, 30.4% to 68% [5-7], than the general population of 10.4% to 23.9% [8-10]. Cough is the major cause of UI among 80 to 90% of females with cystic fibrosis, as frequent coughing may lead to imbalance within the muscles of abdomino-pelvic capsule [11]. The leakage of urine occurs when intra-vesical pressure exceeds urethral closure pressure in the absence of a detrusor contraction, or due to bladder neck hyper-mobility or poor urethral closure pressure [12]. In the literature, focus has been directed to females and coughing whereas investigation on incontinent males is scant [1]. A recent population-based study in the USA reported that men with asthma had twice the odds of leakage, especially for White men [13]. The purpose of this study is to ascertain the relationship between UI and respiratory symptoms in middle-aged and older community-dwelling men. The finding should be beneficial for the prevention and treatment of this irritating condition.

## Methods

### Subjects

Seven hundred community-dwelling men aged 40 years or above were recruited in middle and southern Japan. This convenience sample of subjects was interviewed at shopping malls or when they attended community centres or undertook health checks at hospital clinics. Subjects were excluded if they had dementia or other health conditions that prohibited them from answering the questions. A total of 668 eligible men were available for analysis after excluding participants with missing demographic details or incomplete screening tests of respiratory symptoms. No statistically significant differences were found between the included and the excluded subjects in terms of clinical and other variables. The purpose and procedure were explained to each participant before obtaining their written consent. Confidentiality and the right to withdraw without prejudice were ensured and maintained throughout the study. Approval of the project protocol was obtained from the Human Research Ethics Committee of the researchers' institution (approval number HR 90/2005).

### Questionnaires

A structured questionnaire incorporating the International Consultation on Incontinence Questionnaire-Short Form (ICIQ-SF) [14] was administered face-to-face to assess UI status and to collect demographic information. The ICIQ-SF is a measure for evaluating the severity of urinary loss and condition-specific quality of life. The reliability, validity, and sensitivity of the instrument have been established [14,15]. The questionnaire consists of three components to determine frequency, quantity, and impact of urine leakage. UI was defined as a minimal amount of leakage of at least "once a week or less often", i.e. any urine leakage, during the past year. An additional question was added to the ICIQ-SF to find out how long the person had the condition.

Two screening instruments, Medical Research Council's *dyspnoea *scale [16] and the Australian Lung Foundation's *Feeling Short of Breath *scale [17], were used to assess respiratory symptoms of each individual. The latter scale consisted of five simple questions: (i) Do you cough several times most days? (ii) Do you bring up phlegm or mucous most days? (iii) Do you get out of breath more easily than others your age? (iv) Are you over 40 years old? (v) Are you smoker or ex-smoker?

Information on health conditions including hypertension, ischemic stroke, diabetes mellitus, depression and cancer was solicited from the participants. A further question on 'life-long physical activity involvement' was appended to the questionnaire, defined as "doing active sports or vigorous exercise long enough to get sweaty, at least twice a week", over the entire life course [18]. The 5-level categorical variable was dichotomized to 'not active' ("never been much involved" to "intermittently involved") and 'active' ("always been involved").

### Statistical analysis

Characteristics of the two groups (with and without UI) were compared using chi-square test for categorical variables and *t *test for continuous variables. Logistic regression analyses were then performed to estimate the prevalence of UI in relation to respiratory symptoms. Both crude and adjusted odds ratios were obtained as estimates of relative risk, the latter accounted for the effects of confounding variables namely age, body mass index (BMI), smoking, alcohol drinking status and the presence of co-morbidities.

## Results

For the 668 participants, their average age was 62.7 (standard deviation (SD) 7.5; range 43 to 82) years and mean BMI was 23 (SD 3.1; range 14.5 to 37.8) kg/m^2^. Most of the men were married (87.6%) and 35% of them had retired. A substantial proportion of subjects smoked (26.3%) and consumed alcohol (70.7%) on at least a monthly basis.

The prevalence of UI was 7.6%. Table 1 presents the characteristics of subjects by UI status. The incontinent men were three years older on average and had significantly higher prevalence of respiratory symptoms than those without the condition, especially for breathlessness (45% versus 30%, *p *= 0.025). The binary breathlessness outcome was validated against the Medical Research Council's dyspnoea score. The observed spearman rank correlation of 0.7 confirmed a good agreement between the two scales. The observed dyspnoea score was also higher among the incontinent men, but the two groups were similar with respect to other co-morbidities and physical activity involvement.

**Table 1:** Characteristics of subjects by urinary incontinence status (n = 668)

Characteristic	With UI (n = 51)	Without UI (n = 617)	*p*
**Age **(years)	65.4 (SD 7.2)	62.4 (SD 7.5)	0.007
**Body mass index **(kg/m^2^)	22.9 (SD 4.1)	23.0 (SD 3.0)	0.792
**Dyspnoea score**	1.12 (SD 1.11)	0.84 (SD 1.12)	0.032
**Breathlessness**	22 (44.9%)	178 (29.6%)	0.025
**Cough**	13 (26.5%)	92 (15.3%)	0.040
**Sputum**	18 (36.7%)	180 (29.9%)	0.317
**Smoking pack-years**	48.6 (SD 27.3)	45.8 (SD 27.2)	0.513
**Smoking status^1^**			0.806
never smoker	9 (17.6%)	100 (26.0%)	
ex-smoker	27 (52.9%)	352 (57.6%)	
current smoker	15 (29.4%)	159 (16.4%)	
**Alcohol drinking**			0.036
yes	30 (59%)	440 (72%)	
no	21 (41%)	177 (28%)	
**Marital status^1^**			0.124
single/divorced	9 (18%)	68 (11%)	
married	42 (82%)	543 (89%)	
**Retirement status**^1^			0.092
retired	23 (45%)	212 (35%)	
not retired	28 (55%)	399 (65%)	
**Physical activity**^1^			0.336
not active	40 (78.4%)	441 (72.2%)	
active	11 (21.6%)	170 (27.8%)	
**Co-morbidities**			
Hypertension	7 (13.7%)	139 (22.7%)	0.137
Diabetes mellitus	7 (13.7%)	58 (9.5%)	0.372
Cancer	3 (5.9%)	22 (3.6%)	0.410
Ischemic stroke	0 (0%)	17 (2.8%)	0.228
Depression	0 (0%)	9 (1.5%)	0.383

^1 ^missing data present

UI, urinary incontinence.

SD, standard deviation.

Table 2 summarises the ICIQ-SF outcomes. Urine leakage among the 51 incontinent subjects was typically "a small amount" (84.3%) and occurred once a week or less often (58.8%). The mean ICIQ-SF total score was 6 (SD 2.7) and no one considered the condition to have interfered with their daily life to a great extent. The most common occurrence of urine loss was before reaching the toilet (number (n) = 33, 64.7%). While 30 (58.8%) of the incontinent men experienced urge incontinence, six (11.8%) sustained stress-type leakage, two (3.9%) had mixed and 13 (25.5%) reported other types. On average they had urine leakage for 2.5 (SD 1.8) years with a maximum duration of 10 years.

**Table 2:** Characteristics of incontinent men (n = 51)

ICIQ item	n	%
**Frequency of leakage**		
About once a week or less often	30	58.8
Two or three times a week	11	21.6
About once a day	4	7.8
Several times a day	6	11.8
All the time	0	0
**Quantity of urine loss**		
Nothing	1	2.0
A small amount	43	84.3
A moderate amount	5	9.8
A large amount	2	3.9
**Interfere with everyday life**	Mean 2.0	SD 1.9
0 (not at all)	15	29.4
1-3	28	54.9
4-7	8	15.7
8-10 (a great deal)	0	0
**When urine leakage occurs **(multiple responses)		
before reaching toilet	33	64.7
cough or sneezing	2	3.9
asleep	2	3.9
physically active/exercising	6	11.8
after urinating and dressed	10	19.6
others	2	3.9
**ICIQ score **(range 0 to 21)	Mean 6.0	SD 2.7

ICIQ, International Consultation on Incontinence Questionnaire.

SD, standard deviation.

The proportion of men affected by UI increased from 6.4% without to 11% with breathlessness. Results of logistic regression analyses are given in Table 3, which confirmed all three respiratory symptoms were associated with increase in prevalence of UI. In particular, the adjusted odds ratio (OR) of 2.11 (95% confidence interval (CI) 1.10-4.06) was significant for breathlessness, even after accounting for individual's age, BMI, smoking and alcohol drinking status. Moreover, none of the five health conditions were found to be significant, while the effect of breathlessness was not modified by the inclusion of these co-morbidities into the multivariate model. The correlation between the ICIQ score and the dyspnoea score was 0.24. However, the dose-response effect of dyspnoea on UI was not significant (adjusted OR = 1.17, 95% CI 0.90- 1.51).

**Table 3:** Respiratory symptoms and risk of urinary incontinence

Respiratory symptom	Distribution	Crude odds ratio			Adjusted odds ratio^1^		
	n (%)	Odds ratio	95% CI	*p*	Odds ratio	95% CI	*p*
**Breathlessness**				0.027			0.025
No	455 (68.1%)	1			1		
Yes	201 (30.1%)	1.94	(1.08, 3.50)		2.11	(1.10, 4.06)	
**Cough**				0.043			0.137
No	551 (82.5%)	1			1		
Yes	105 (15.7%)	2.00	(1.02, 3.92)		1.73	(0.84, 3.54)	
**Sputum**				0.319			0.423
No	457 (68.4%)	1			1		
Yes	199 (29.8%)	1.36	(0.74, 2.50)		1.31	(0.68, 2.51)	

^1 ^adjusted for age, BMI, smoking and alcohol drinking status in logistic regression model

CI, confidence interval.

## Discussion

This study provides the first report of the relationship between UI and respiratory symptoms among middle-aged and older men. Breathlessness was found to be associated with UI. A cross-sectional study of 7,947 community-dwelling women in the USA reported that faster gait speed (≥ 1.04 meter/second) was related to decreased daily UI (OR 0.8, 95% CI 0.6-1.0) compared with lower gait speed (< 0.86 meter/second) [19]. Although walking slowly may not necessarily imply breathlessness, people who can walk fast are unlikely to suffer from breathlessness.

For women with cystic fibrosis [11] and asthma [13], stress incontinence has been related to their chronic coughing and sneezing which raise intra-abdominal pressure and cause damage to the pelvic floor [13]. Unlike women, urge-type leakage was most common among our sample of incontinent men. The observed association with breathlessness can be attributed to the continence mechanism, especially neurological control of the pelvic floor and micturation [11]. The nerve supply of the pelvic floor is via the pudendal nerve, as part of the somatic nervous system. Bladder filling and storage of urine involves autonomic control via the sympathetic system and bladder voiding is under parasympathetic control [11]. Meanwhile, breathlessness is under the sympathetic system. People who feel breathlessness are more likely to have an imbalance between sympathetic system and parasympathetic control, or may have less parasympathetic control. Once leakage occurs there is a chronic and progressive deterioration of the pelvic neuromuscular function [5].

Some limitations of the study should be considered. Classification of UI status was based on self report via the ICIQ-SF rather than objective measurements of urine loss. Despite the subjective perception of the condition, it is now recognised that the use of psychometrically robust self-completion questionnaires is a valid approach for assessing UI. The ICIQ-SF has good measurement properties and encompasses all aspects of incontinence [14,15,20]. Moreover, face-to-face interviews were conducted by the same investigator to avoid misinterpretation of the questions and to reduce inter-interviewer bias. All participants resided in the community and should be representative of the underlying population. Selection bias might still exist because these voluntary participants were not randomly selected. The observed association could be confounded by the presence of co-morbid conditions. The lack of confirmation of other co-morbidities such as benign prostatic hypertrophy was another limitation of the study. Physical activity and several self-reported health conditions were not associated with urine leakage and thus unlikely to be confounders. Nevertheless, population-based prospective cohort studies are needed to confirm the apparent cause-and-effect relationship between UI and breathlessness. Experimental research to further understand the underlying biological mechanism is also important.

## Conclusions

The present study has highlighted the additional burden that breathlessness can impose on middle-aged and older men. Education and regular assessment for UI are recommended, and treating respiratory symptoms should become part of the routine health management of older adults to prevent the development of this distressing condition.

## Competing interests

The authors declare that they have no competing interests.

## Authors' contributions

FH collected the data and drafted the manuscript. AHL analysed the data and revised the manuscript. TH and YT assisted with data collection and interpretation of the findings. All authors read and approved the final version of the paper.

## Pre-publication history

The pre-publication history for this paper can be accessed here:

http://www.biomedcentral.com/1471-2466/10/2/prepub

## References

[B1] Hunskaar S Burgio K Diokno AC Herzog AC Hjälmaás K Lapitan MC Eds Abrams P, Cardozo L, Khoury S, Wein A Incontinence: Second International Consultation on Incontinence Paris, July 1-3, 2001 2002 2 Plymouth: Health Publication Ltd 182 191

[B2] Dallosso HM McGrother CW Matthews RJ Donaldson MM The association of diet and other lifestyle factors with overactive bladder and stress incontinence: a longitudinal study in women BJU Int 2003 92 69 77 10.1046/j.1464-410X.2003.04271.x 12823386

[B3] Danforth KN Townsend MK Lifford K Curhan GC Resnick NM Grodstein F Risk factors for urinary incontinence among middle-aged women Am J Obstet Gynecol 2006 194 339 345 10.1016/j.ajog.2005.07.051 16458626 PMC1363686

[B4] Yoshimura K Kamoto T Tsukamoto T Oshiro K Kinukawa N Ogawa O Seasonal alterations in nocturia and other storage symptoms in three Japanese communities Urology 2007 69 864 870 10.1016/j.urology.2007.01.037 17482923

[B5] Orr A McVean RJ Webb AK Dodd ME Questionnaire survey of urinary incontinence in women with cystic fibrosis BMJ 2001 322 1521 10.1136/bmj.322.7301.1521 11420273 PMC33391

[B6] Cornacchia M Zenorini A Perobelli S Zanolla L Mastella G Braggion C Prevalence of urinary incontinence in women with cystic fibrosis BJU Int 2001 88 44 48 10.1046/j.1464-410x.2001.02242.x 11446844

[B7] Moran F Bradley JM Boyle L Elborn JS Incontinence in adult females with cystic fibrosis: a Northern Ireland survey Int J Clin Pract 2003 57 182 183 12723720

[B8] Barry MJ Link CL McNaughton-Collins MF McKinlay JB Overlap of different urological symptom complexes in a racially and ethnically diverse, community-based population of men and women BJU Int 2008 101 45 51 17868419 10.1111/j.1464-410X.2007.07191.x

[B9] Jackson RA Vittinghoff E Kanaya AM Miles TP Resnick HE Kritchevsky SB Simonsick EM Brown JS Urinary incontinence in elderly women: findings from the Health, Aging, and Body Composition Study Obstet Gynecol 2004 104 301 307 15292003 10.1097/01.AOG.0000133482.20685.d1

[B10] Kocak I Okyay P Dundar M Erol H Beser E Female urinary incontinence in the west of Turkey: prevalence, risk factors and impact on quality of life Eur Urol 2005 48 634 641 10.1016/j.eururo.2005.04.017 15963633

[B11] Dodd ME Langman H Urinary incontinence in cystic fibrosis J R Soc Med 2005 98 Suppl 45 28 36 16025765 PMC1308806

[B12] Neumann PB Grimmer KA Deenadayalan Y Pelvic floor muscle training and adjunctive therapies for the treatment of stress urinary incontinence in women: a systematic review BMC Womens Health 2006 6 1 28 10.1186/1472-6874-6-11 16805910 PMC1586224

[B13] Tennstedt SL Link CL Steers WD McKinlay JB Prevalence of and risk factors for urine leakage in a racially and ethnically diverse population of adults: the Boston Area Community Health (BACH) Survey Am J Epidemiol 2008 167 390 399 10.1093/aje/kwm356 18182376

[B14] Avery K Donovan J Peters TJ Shaw C Gotoh M Abrams P ICIQ: a brief and robust measure for evaluating the symptoms and impact of urinary incontinence Neurourol Urodyn 2004 23 322 330 10.1002/nau.20041 15227649

[B15] Karantanis E Fynes M Moore KH Stanton SL Comparison of the ICIQ-SF and 24-hour pad test with other measures for evaluating the severity of urodynamic stress incontinence Int Urogynecol J Pelvic Floor Dysfunct 2004 15 111 116 10.1007/s00192-004-1123-2 15014938

[B16] Bestall JC Paul EA Garrod R Garnham R Jones PW Wedzicha JA Usefulness of the Medical Research Council (MRC) dyspnoea scale as a measure of disability in patients with chronic obstructive pulmonary disease Thorax 1999 54 581 586 10377201 10.1136/thx.54.7.581 PMC1745516

[B17] Managament tools http://www.copdx.org.au/checklist/index.asp

[B18] O' Brien Cousins S Tan M Sources of efficacy for walking and climbing stairs among older adults Phys Occup Ther Geriatr 2002 20 51 68

[B19] Brown JS Seeley DG Fong J Black DM Ensrud KE Grady D Urinary incontinence in older women: who is at risk? Study of Osteoporotic Fractures Research Group Obstet Gynecol 1996 87 715 721 10.1016/0029-7844(96)00013-0 8677073

[B20] Avery KN Bosch JL Gotoh M Naughton M Jackson S Radley SC Valiquette L Batista J Donovan JL Questionnaires to assess urinary and anal incontinence: review and recommendations J Urol 2007 177 39 49 10.1016/j.juro.2006.08.075 17161997

